# Tendency to overeat predicts an elevated body mass index trajectory across school-age years

**DOI:** 10.1038/s41598-025-90786-7

**Published:** 2025-02-22

**Authors:** Catharina Sarkkola, Sohvi Lommi, Kris Elomaa, Eero Kajantie, Satu Männistö, Heli Viljakainen

**Affiliations:** 1 Folkhälsan Research Center, Helsinki, Finland; 2 Department of Public Health, Faculty of Medicine, University of Helsinki, Helsinki, Finland; 3 Faculty of Medicine, University of Helsinki, Helsinki, Finland; 4 Finnish Institute for Health and Welfare (THL), Helsinki, Finland; 5 Medical Research Center Oulu, Oulu University Hospital and University of Oulu, Oulu, Finland; 6 Clinical Medicine Research Unit, University of Oulu, Oulu, Finland; 7 Department of Clinical and Molecular Medicine, Norwegian University of Science and Technology, Trondheim, Norway; 8 Folkhälsan Research Center, Topeliuksenkatu 20, 00250 Helsinki, Finland

**Keywords:** Nutrition, Paediatric research, Epidemiology, Weight management, Risk factors, Epidemiology, Paediatric research

## Abstract

Overeating is a complex appetite trait, cross-sectionally linked to an elevated weight in children. However, little is known about longitudinal associations. Therefore, we studied how a tendency towards overeating predicts weight development between 8 and 16 years of age. In this study among 4517 children from the Finnish Health in Teens cohort, parents reported their child’s tendency to overeat when children were on average 11.2 (SD 0.8) years old. Children were then categorised as overeating, possibly overeating, or not overeating. Height and weight measurements from two data collection periods were combined with growth data from a national health register, and age- and sex-standardised body mass index z-scores (BMIz) were calculated using the International Obesity Task Force reference. Children also reported their lifestyle factors, including food consumption, physical activity, screen time, and sleep patterns. We examined the association between overeating and BMIz using a linear mixed model, adjusting for age, sex, specific food consumption frequencies, physical activity, screen time, and sleep duration. We further analysed whether associations differed by age, food consumption frequencies, or physical activity. The average BMIz in the overeating group was 1.18 (95% CI 1.10─1.26) units higher compared with those without overeating, but remained stable as age increased. Among those without overeating, BMIz increased 0.043 units per year of age (*p* < 0.001). Physical activity, but not food consumption, modified the association between overeating and BMIz (*p* for interaction = 0.038). In the lowest third of physical activity (≤ 5 h/week), BMIz was 1.28 units higher (95% CI 1.15─1.41) in the overeating compared with the no overeating group, while in the highest third (≥ 9 h/week) the effect size was 1.08 (95% CI 0.93─1.24). In conclusion, children with a parent-reported tendency to overeat exhibited an elevated, but stable mean BMIz across adolescence. Public health programmes tackling the obesity epidemic should consider the differences in appetite self-regulation among children.

**Supplementary Information:**

The online version contains supplementary material available at 10.1038/s41598-025-90786-7.

## Introduction

Eating beyond one’s nutritional need—or overeating—is a complex physiological and psychological process influenced by genetics, environment, individual behaviour, and neural processes^1,2^. Overeating can result from either a low satiety responsiveness (SR), which refers to a low sensitivity to internal fullness cues, or alternatively or in conjunction with a high food responsiveness (FR) due to high biological, cognitive, and emotional responses to food cues including emotional overeating (EOE)^2^. Overeating and its different characteristics in children are typically measured through parent-reported questionnaires.

In a large UK study (*n*= 4844), an overeating trajectory between the ages of 1 and 9 years was associated with a higher body mass index z-score (BMIz) at 11 years of age^3^. In that study, parents reported whether their child overate to a worrying degree. Canadian children (*n*= 1498) reported to overeat in general between 2 and 4 years of age were more likely to live with overweight at 4 years of age than children who never overate^4^. In the same cohort, overeating at 4 years of age predicted a high, yet stable BMIz trajectory between ages 4 and 10^5^.

According to a review and meta-analysis by Kininmonth et al.^6^, several cross-sectional studies across continents have consistently shown that the different characteristics of overeating (low SR, high FR and high EOE) are linked to measures of adiposity in children with a broad age range. By contrast, longitudinal studies of these associations remain limited, with varied methodologies and inconsistent results regarding the various aspects of overeating. Studies from Norway (*n*= 675)^7^ and China (*n*= 2566)^8^ investigating BMIz changes observed that only a high FR and low SR in girls, respectively, associated with an increase in BMIz. The baseline ages were 6 and 11─17 years with follow-up periods of 2 and 1.5 years, respectively. Studies from Portugal (*n*= 4264)^9^ and Norway (*n*= 802)^10^ found no associations between any characteristic of overeating with a BMIz change from ages 7 to 10 and 8 to 14 years, respectively.

Given the high prevalence of childhood obesity in Finland and worldwide^11,12^, as well as its link to an increased risk of numerous non-communicable diseases, it is important to identify the contribution of overeating to weight development. Specifically, studies with older children, extended follow-up periods and multiple BMI measurements remain sparse. Therefore, this study primarily aimed to examine the association between a tendency towards overeating (hereafter, simply ‘overeating’) and BMIz, and to explore the potential interaction with age in 8–16-year-old Finnish children. The secondary aim was to investigate whether the association between overeating and BMIz differs by fruit and vegetable, sugary product, or fast food consumption frequency, or leisure-time physical activity.

## Methods

### Study population

This study included participants from the prospective Finnish Health in Teens study (Fin-HIT) covering Finland’s nine largest cities and their surrounding areas^13^. This cohort was recruited in 2011–2014, mainly through schools, and initially consisted of 11 407 children aged 8–13 years and their 6046 parents, 87% of them mothers. In this study, we included a sample of 4517 children for whom data were available on height and weight, parent-reported overeating, and lifestyle factors. Data on children’s age and sex were confirmed through linkage to the National Population Information System at the Population Register Centre.

This research adhered to the Declaration of Helsinki and the study protocol was approved by the Coordinating Ethics Committee of the Hospital District of Helsinki and Uusimaa (169/13/03/00/10). Written informed consent was obtained from the children and one parent per child.

### Overeating

At baseline, we assessed overeating using a question adapted from the Avon Longitudinal Study of Parents and Children (ALSPAC, UK)^14^, a parent-administered questionnaire at 128 months. Parents were asked to complete the question *Do you agree or disagree with this statement: ‘If I did not guide or regulate my child’s eating*,* s/he would eat too much.’* Answers were scored on a five-point Likert scale: *1*,* agree (n = 111; 2.5%); 2*,* slightly agree (n = 345; 7.6%); 3*,* neither agree nor disagree (n = 185; 4.1%); 4*,* slightly disagree (n = 532; 11.8%); and 5*,* disagree (n = 3344; 74.0%)*. Due to the small number of responses, we combined the first two response options. Similarly, we combined the last two response options, creating a variable with three categories: overeating, possible overeating, and no overeating.

### Anthropometric measurements

**Fig. 1:**
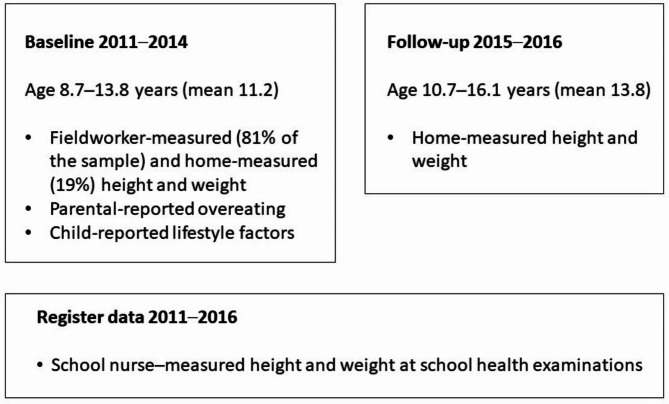
Flowchart of the Fin-HIT study, data shown for the final sample (*n* = 4517).

We used anthropometric measurements from two Fin-HIT data collection periods (baseline and first follow-up) and from a national register, as shown in Fig. 1. At baseline, trained fieldworkers measured children’s height and weight at school^13^. Those children participating at home (19%) provided self-reported measurements taken with an adult’s assistance. They received detailed written instructions and a measuring tape, and were asked to weigh themselves using scales. During follow-up, all participants were asked to self-report the measurements in a similar manner at home. In our previous validation study, we reported that the difference between home-measured and objectively measured mean BMI was only marginal^15^. Thus, these self-reported measurements were proven valid for epidemiological studies. In addition, we obtained measured height and weight data collected between 2011 and 2016 from the national Register of Primary Health Care visits (Avohilmo), maintained by the Finnish Institute for Health and Welfare^16^. This register covers growth measurements from health examinations at schools, taken by a school nurse.

Based on height and weight, we calculated BMIz according to the International Obesity Task Force (IOTF) age- and sex-specific reference system^17^. We integrated BMIz values from both data sources to construct BMIz trajectories spanning from 6 to 18 years of age. Because different measurement protocols may contribute to variation in BMIz values, we evaluated the clinical relevance of any differences between the register and Fin-HIT BMIz values statistically to ensure their comparability (as described in the supplementary material; *Supplementary Figs. 1*,* 2*,* 3*,* 4*). We concluded that BMIz calculated in these two data sources were comparable with a low bias and, therefore, merged them for the data analysis.

For the final dataset, we excluded BMIz measurements prior to baseline data collection in order to analyse the prospective association of overeating with BMIz trajectory. We included children with at least two BMIz measurements regardless of data source. Since there was only one child with a measurement after 16 years, we included measurements taken when individuals were aged 8 to 16 years in the final sample. Altogether, the data then consisted of 14 895 BMIz measurements, which on average consisted of 3.3 measurements per child. Of the children, 49% had two BMIz measurements, with similar proportions across all three groups: overeating (47%), possible overeating (51%), and no overeating (49%).

### Covariates

Information on several lifestyle factors was collected at baseline through questionnaires. The children completed a 16-item food propensity questionnaire adapted from the World Health Organization’s Health Behaviour in School-Aged Children Study^18^. The consumption frequency of each food item during the previous month was rated on a seven-point scale from ‘not at all’ to ‘several times a day’. Six items represented sugary products: chocolate and sweets, biscuits and cookies, sweet pastry, ice cream, sugary juice drinks, and sugary soft drinks. Based on these items, we calculated the sum frequency of the weekly sugary food consumption, the sweet treat index^19^. The total index score ranged from 0 to 84, with a higher score indicating a higher consumption frequency. In the same way, fresh vegetables, cooked vegetables, and fruit or berries constituted the plant consumption index^20^, while pizza, hamburger or hot dog, and salty snacks comprised the fast food index. Both of these index total scores ranged from 0 to 42.

In addition, the children reported their leisure-time physical activity (in hours per week) and screen time (watching TV or videos, using a computer, playing TV games, etc.; in hours per day on weekdays and weekend days)^21^. We calculated the weighted average of daily screen time by counting five weekdays and two weekend days. Finally, the children provided their bed and wake-up times, respectively, on weekdays and weekend days, to then calculate the weighted average of daily sleep hours.

The parental questionnaire included a question on their highest level of education completed. We recoded the original six-category variable into four groups: 1, comprehensive school, vocational school, or matriculation examination; 2, technical institute qualification; 3, university of applied sciences; and 4, university. We selected the above-mentioned covariates, adding them as confounders in the model based on previous literature. According to several reviews, child lifestyle factors such as diet quality, sleep and screen time are associated with both eating behaviour and weight^22–24^.

### Statistical analyses

We compared the covariates between the three overeating groups (overeating, possible overeating, and no overeating) using the χ^2^test for categorical variables. For normally distributed continuous variables, we presented means and used the analysis of variance (ANOVA). For non-normally distributed continuous variables, we presented medians and used the Kruskal-Wallis test. Normality assessment was based on histograms, and the skewness and kurtosis values^25^.

We tested the association between overeating and BMIz using a linear-mixed model, showing the difference in mean BMIz for all ages between the three overeating groups. We applied the random slope and intercept model, since it had a better explanatory power than random intercept only based on the Akaike Information Criterion^26^. Results are reported as the mean with the 95% confidence interval (CI). We applied three models, the first of which included age and sex. Model 2—which we considered our primary model—was also adjusted for sweet treat, plant consumption, and the fast food index, physical activity, screen time, and sleep duration. Model 3 also included parental education.

In addition, we tested whether the association between overeating and BMIz differs by age—that is, if overeating predicts BMIz development over time by adding an interaction term. Similarly, we tested whether the association differed according to sweet treat, plant, and fast food consumption frequency as well as by level of physical activity. For the interaction analyses, we applied model 2. We ran additional subgroup analyses for each physical activity third: ≤5 h/week, 6─8 h/week, and ≥ 9 h/week. We performed all statistical analyses using the software package IBM SPSS Statistics version 29.0. We set the level of statistical significance to *p* < 0.05.

## Results

### Participant characteristics

This study included a total of 4517 children. The mean (standard deviation, SD) age at baseline was 11.2 (0.8) years. Across the entire sample, girls constituted 51%, whereas in the overeating group, they comprised only 45% *(*Table 1*).* The children in the overeating group reported less physical activity, more screen time, and less frequent consumption of sweet treats than those with no overeating. By contrast, the consumption frequency of plants and fast food, or sleep duration did not differ across groups.

**Table 1:** Child and parental characteristics at baseline in the entire sample and by overeating categories reported as mean (SD) for continuous variables unless noted otherwise and n (%) for categorical variables.

	Total	Overeating	Possible overeating	No overeating	ANOVA
	*n* = 4517	*n* = 456	*n* = 185	*n* = 3876	*p* value
Child characteristics					
Sex					0.021^a^
Girls	2314 (51.2)	207 (45.4)	90 (48.6)	2017 (52.0)	
Boys	2203 (48.8)	249 (54.6)	95 (51.4)	1859 (48.0)	
Age at baseline, in years	11.2 (0.8)	11.2 (0.7)	11.1 (0.7)	11.2 (0.8)	0.384^b^
*Food consumption frequency*,* times/week*					
Sweet treat index^c^	6.5 (7.0)	5.5 (7.0)	6.5 (8.0)	7.0 (7.0)	0.002^d^
Plant consumption index	13.5 (8.6)	12.8 (8.6)	12.6 (8.1)	13.6 (8.6)	0.053
Fast food index^c^	1.5 (1.0)	1.5 (1.0)	1.5 (1.0)	1.5 (1.0)	0.377^d^
Physical activity, h/week	6.7 (2.7)	6.2 (2.7)	6.7 (2.8)	6.7 (2.7)	< 0.001
Screen time, h/day	3.2 (2.0)	3.6 (2.2)	3.6 (2.6)	3.1 (2.0)	< 0.001^b^
Sleep duration, h/night	9.7 (0.8)	9.7 (0.7)	9.6 (0.8)	9.7 (0.8)	0.466
Parental characteristics					
Education^e^					0.086^a^
Maximum secondary education^f^	915 (20.7)	102 (22.8)	52 (28.9)	761 (20.1)	
Technical institute qualification	1009 (22.8)	106 (23.7)	37 (20.6)	866 (22.8)	
Diploma from a university of applied sciences	814 (18.4)	72 (16.1)	32 (17.8)	710 (18.7)	
Academic or university degree	1680 (38.0)	167 (37.4)	59 (32.8)	1454 (38.4)	

^a^χ^2^

^b^Brown-Forsythe test for groups with unequal variances

^c^Median (interquartile range)

^d^Kruskal-Wallis test

^e^Missing, *n* = 99

^f^Comprehensive school, vocational school, or matriculation examination

### Association between overeating and BMIz

**Fig. 2:**
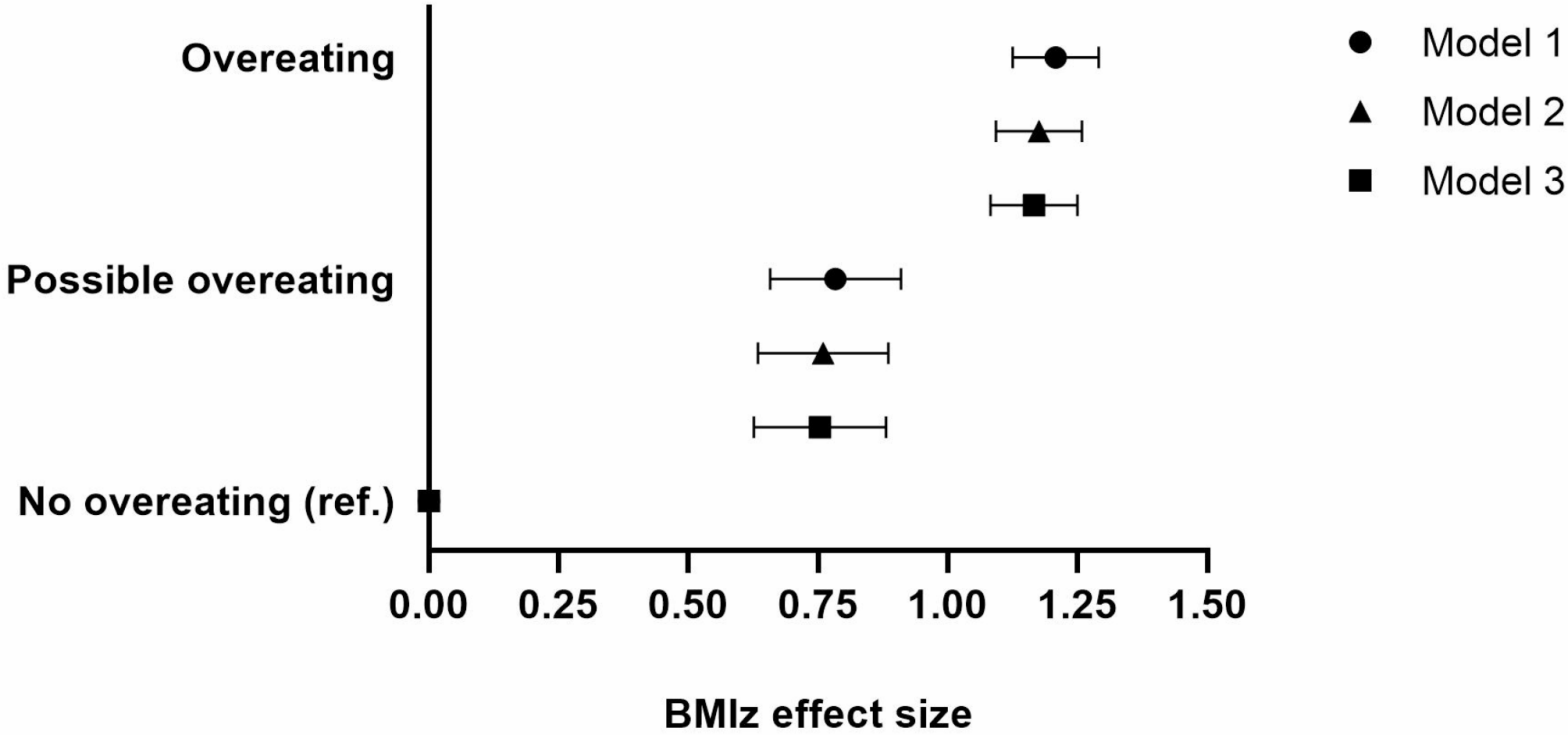
Comparison of BMIz effect sizes between the overeating categories, parameter estimates, and 95% confidence intervals. Adjustments: model 1 adjusted for age and sex; model 2 also includes the sweet treat, plant consumption, and fast food index, as well as physical activity, screen time, and sleep duration; and model 3 also included parental education.

The average BMIz for the entire age range differed between the three overeating groups (*p* < 0.001; Fig. 2). In the primary model (model 2), the overeating group had a 1.18-units (95% CI 1.10─1.26) and the possible overeating group had a 0.76-unit (95% CI 0.64─0.89) higher BMIz than those without overeating. Adjusting for parental education in model 3 did not change the results.

**Fig. 3:**
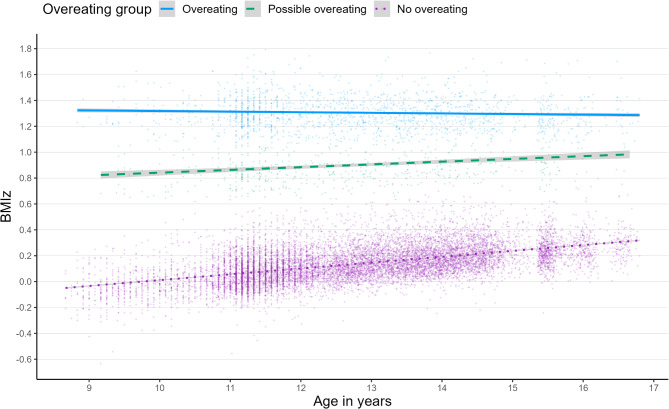
Changes in BMIz with increasing age in the three overeating categories: overeating (*n* = 456), possible overeating (*n* = 185), no overeating (*n*= 3876). The BMIz range for normal weight is −0.975 to 1.244 for girls and −1.014 to 1.310 for boys^17^.

In the entire sample, the increase in BMIz per year of age was 0.036 units (*p* < 0.001) in all models. The BMIz differences between the three groups flattened with an increasing age: in the overeating group, BMIz decreased by 0.010 units per year of age (*p* = 0.132), while among those without overeating, BMIz increased by 0.043 units per year (*p* < 0.001 for model 2; Fig. 3). In the possible overeating group, BMIz increased by 0.012 units per year (*p* = 0.264). The age slopes among the overeating versus no overeating groups and possible overeating versus no overeating groups differed from each other (*p* for interaction < 0.001 and < 0.006, respectively).

**Fig. 4:**
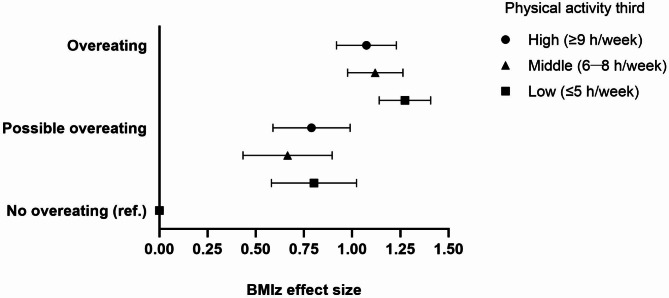
Comparison of BMIz effect sizes between the overeating categories in the physical activity thirds, parameter estimates, and 95% confidence intervals. Adjustments according to model 2: age, sex, sweet treat, plant consumption, and fast food indexes, screen time, and sleep duration.

Neither the consumption frequency of sweet treats, plants nor fast foods modified the association between overeating and BMIz (*p* for interaction: 0.532, 0.114, and 0.571, respectively). By contrast, leisure-time physical activity modified the association (*p* for interaction = 0.038). The subgroup analyses were in line with the interaction analysis *(*Fig. 4*).* In the lowest third of physical activity (≤ 5 h/week), the association between overeating and BMIz was 1.28 (95% CI 1.15─1.41), 1.12 (95% CI 0.98─1.27) in the middle third (6─8 h/week), falling to 1.08 (95% CI 0.93─1.24) in the highest third (≥ 9 h/week).

## Discussion

In this prospective cohort study of approximately 4500 Finnish children, we found that a parent-reported tendency towards overeating associated with a higher and more stable BMIz trajectory across school-aged children compared with children with no overeating tendency. However, the differences between groups flattened with age. We combined BMIz measurements from our own data collection using measurements from a national health register, allowing us to construct trajectories using several timepoints between the ages of 8 and 16 years. This is the first study to show an association between overeating and weight development during the critical years of adolescent growth.

Our findings agree with a previous Canadian study in which children with parent-reported overeating in general were more likely to exhibit a high, but stable BMIz trajectory^5^. However, those children were followed at a younger age, between the ages of 4 and 10 years. Our findings also mirror previous prospective studies showing that some aspects of overeating (low SR, high FR or high EOE) associate with a higher BMIz^6^. However, the few studies on subsequent weight change yielded inconclusive results. Several studies found no links between the different components of overeating and BMIz change^7–10^. Among Norwegian children, however, a high FR at age 6 predicted an increase in BMIz until 8 years of age^7^, and, among Chinese adolescent girls, a lower SR compared with a higher SR associated with larger increases in BMIz over a 1.5-year time period^8^, contrasting our findings. The use of different questionnaires to evaluate eating behaviours may complicate comparisons of the findings. The Chinese study used the Chinese version of the Adult Eating Behaviour Questionnaire^27^, while the other studies used the Children’s Eating Behaviour Questionnaire^28^.

In our study, children with overeating had on average a 1.18-unit higher BMIz than those without overeating and their mean BMIz remained rather stable and highest throughout the follow-up period. The difference in 1.18 BMIz units correspond to 9.2 kg of weight for an 11-year-old, 150-cm-tall child. However, the largest differences between groups were observed at the youngest ages. The groups converged due to an increase in the mean BMIz among those without overeating.

Our finding that mean BMIz did not further increase among children with overeating after baseline could indicate that previously important appetite traits played a smaller role in obesity development as a child grows. We hypothesise that overeating or low satiety responsiveness stimulates weight gain in early childhood but BMIz stabilises before adolescence. Several studies on BMI trajectories in childhood have identified stable high BMI trajectory groups^5,29,30^. This finding might also result from an adult or the child her/himself restricting and other weight control practices among those children living with overweight or obesity. The prevalence of overweight tends to increase during childhood and adolescence, as shown by Finnish data among 2─16-year-olds^11^. Our finding regarding children without overeating agrees with this observation.

The consumption frequency of fruits and vegetables, sugary products, or fast food did not modify the association between overeating and BMIz. By contrast, the association differed depending upon the amount of leisure time physical activity. Children falling within the lowest third for physical activity exhibited the largest effect size for BMIz, while those in the highest third exhibited the smallest effect size. These findings are logical and reflect a possible compensatory behaviour since a high amount of physical activity appears to mitigate the effect of overeating on BMIz^31–33^. There is also the possibility that physical activity increases appetite in these children, leading their parents to classify them as overeaters, even though parents may understand that more physical activity requires a higher energy intake.

Appetite self-regulation, including SR and FR, may decline during early childhood^34^. Infants are highly responsive to internal hunger and satiety cues, although considerable inter-individual variability exists, likely being genetically determined^6,35^. With age the ability to self-regulate food intake decreases. External cues become more relevant, rendering older children more vulnerable to overeating and excessive weight^36^. However, previous studies have focused on children under 11 years of age, with the findings on changes in SR and FR inconsistent across studies^3,28,37,38^. In fact, some studies reported only small decreases in the SR subscale and small increases in the FR subscale with age^28,37^, while others showed no difference at all^38^. A more recent study explored the overeating trajectory between ages 1 and 10 years^3^. In fact, 70% of the children in that study were not described as overeaters by their parents at any of the timepoints, while 17% exhibited an increasing overeating trajectory. Less is known about the development of children’s eating behaviour after the age of 11 years. In our study, we measured overeating only at the beginning. We considered overeating exposure stable, although it may fluctuate across time. Moreover, the possibility of a reverse causality exists; a higher BMIz or increased weight gain has been prospectively associated with a lower SR, a higher FR, and a higher EOE in 4─12-year-old children^7,9,10,39,40^. In addition, child obesity had a stronger prospective association with parental concern of overeating than vice versa in a Pan-European cohort^41^. Due to limited data, we were not able to assess the bidirectional relationship between overeating and BMIz.

The main strength of this study lies in the large sample representative of a paediatric population in urban and semi-urban areas of Finland. The findings may be generalized to other affluent Western countries with similar obesity prevalence, particularly those in Northern and Central Europe^42^. The anthropometric measurements in the Fin-HIT study were primarily measured by trained fieldworkers; in addition, we obtained objective register data based on school healthcare measurements, resulting in multiple BMIz measurements per child. BMIz originating from these two data sources were comparable (see supplementary material). This is the first study using such comprehensive data on BMIz during a wide age span in adolescence and taking advantage of register data. Although the Avohilmo register is reliable, it has limited national coverage, reaching just 33% in 2016^11,43^. In comparison, register data were available for 47% of the children in our study.

The primary limitation to our study relates to our single, parent-reported, and unvalidated question on overeating. Our measure also divided children into groups instead of providing a continuous ranking on a scale, such as the Children’s Eating Behaviour Questionnaire with mean scores for appetite subscales^28^. The numbers of children with overeating and possible overeating were comparatively small compared with those without overeating. Moreover, the lifestyle factors were reported at baseline, and we cannot rule out the possibility that these behaviours changed during adolescence. Although we considered multiple confounders in the modelling, we might have missed others, such as parental feeding styles. Thus, the possibility of residual confounding exists.

## Conclusions

In this study, we demonstrated that children with a tendency towards overeating had a higher albeit stable mean BMIz throughout adolescence compared with children without this tendency. However, the differences in BMIz trajectories between groups attenuated with age due to an increase in BMIz in the no overeating group. In addition, our findings imply that a higher amount of physical activity may mitigate the effect of overeating on BMIz, but also alternative explanations may exist. Addressing appetite self-regulation in public health programmes tackling the obesity epidemic is crucial. At child health clinics, families could be provided with information on appropriate portion sizes and how to identify the body’s hunger and satiety signals, for example, by using a visual satiety scale^44^. Fostering a balanced relationship with food, and avoiding emotional eating or eating too fast, should be promoted. Encouraging increased physical activity, especially amongst children with a tendency to overeat, could promote healthier growth. Additionally, using a simple parental questionnaire to monitor eating behaviours could aid early interventions, enhancing the impact of health interventions.

## Electronic supplementary material

Below is the link to the electronic supplementary material.

Supplementary Material 1

## Data Availability

The datasets generated and analysed during the current study are available from the corresponding author on reasonable request.
