# Author Correction: Tendency to overeat predicts an elevated body mass index trajectory across school-age years

**DOI:** 10.1038/s41598-025-97099-9

**Published:** 2025-04-11

**Authors:** Catharina Sarkkola, Sohvi Lommi, Kris Elomaa, Eero Kajantie, Satu Männistö, Heli Viljakainen

**Affiliations:** 1 Folkhälsan Research Center, Helsinki, Finland; 2 Department of Public Health, Faculty of Medicine, University of Helsinki, Helsinki, Finland; 3 Faculty of Medicine, University of Helsinki, Helsinki, Finland; 4 Finnish Institute for Health and Welfare (THL), Helsinki, Finland; 5 Medical Research Center Oulu, Oulu University Hospital and University of Oulu, Oulu, Finland; 6 Clinical Medicine Research Unit, University of Oulu, Oulu, Finland; 7 Department of Clinical and Molecular Medicine, Norwegian University of Science and Technology, Trondheim, Norway; 8 Folkhälsan Research Center, Topeliuksenkatu 20, 00250 Helsinki, Finland

Author Correction: *Scientific Reports* 10.1038/s41598-025-90786-7, published online 22 February 2025

The Competing interests section in the original version of this Article was incorrect.

“EK has received grant support from the Research Council of Finland, Signe and Ane Gyllenberg Foundation, Foundation for Pediatric Research, Sigrid Jusélius Foundation and Novo Nordisk Foundation. HV has received grants from the Foundation for Pediatric Research, Medicinska Understödsföreningen Liv och Hälsa rf and Päivikki and Sakari Sohlberg Foundation to support the study. The funding bodies had no role in the design of the study, collection and analysis of data nor decision to publish. All other authors declare no competing financial interests.”

now reads:

“EK has received grant support from the Research Council of Finland, Signe and Ane Gyllenberg Foundation, Foundation for Pediatric Research, Sigrid Jusélius Foundation and Novo Nordisk Foundation. HV has received grants from the Foundation for Pediatric Research, Medicinska Understödsföreningen Liv och Hälsa rf and Päivikki and Sakari Sohlberg Foundation to support the study. The funding bodies had no role in the design of the study, collection and analysis of data nor decision to publish. All other authors declare no competing financial interests. Open access funded by Helsinki University Library.”

The original Article has been corrected.

