# Supplementary material for: Tendency to overeat predicts an elevated body mass index trajectory across school-age years
Source: Sci Rep. 2025 Feb 22;15:6495. doi: 10.1038/s41598-025-90786-7 (PMC11846909; doi:10.1038/s41598-025-90786-7)
Supplement: Supplementary file 1 — Supplementary Material 1 [file 41598_2025_90786_MOESM1_ESM.pdf]

Sarkkola C, Lommi S, Elomaa K, Kajantie E, Männistö S, Viljakainen H. Tendency to overeat predicts an elevated body mass index trajectory across school-age years

SUPPLEMENTARY FILE: Comparison of two data sources on anthropometric measurements

Different data sources may contribute to variation in BMIz values. We, therefore, evaluated the differences between the Avohilmo register and Fin-HIT datasets. First, we selected all participants with a measurement in each of the three categories: register (n = 8598), Fin-HIT baseline, and Fin-HIT follow-up (n = 4869) to compare their means. This crude comparison of the data sources revealed similarities in the means and standard deviations (SD) despite unequal sample sizes *(Supplementary Figure 1).*

Second, to address the intraindividual differences in BMIz values between the data sources, we conducted a paired data comparison. For this, we selected a subset of participants for whom both a register and a Fin-HIT measurement were collected within six months of one another. Thus, the final subset was reduced to 2439 measurement pairs from 1864 individuals. Again, the mean values of the paired data by age were similar *(Supplementary Figure 2),* and the additional analyses showed that the intraindividual differences remained constant over a six-month time period *(Supplementary Figure 3).*

Finally, we compared the difference in mean values for paired data by age. Paired sample t-tests showed statistically significant differences in the mean BMIz values at ages 11.0, 11.5, 12.0, and 12.5 years only *(Supplementary Figure 4).* The largest significant difference in the paired BMIz was 0.092 in the 11.5-year age group, corresponding either to a 0.95-kg difference in weight or a 0.54-cm difference in height. Such variations fall within normal daily variations (1, 2, 3) and are not clinically relevant.

REFERENCES

1. Siklar Z, Sanli E, Dallar Y, Tanyer G. Diurnal variation of height in children. Pediatr Int. 2005;47(6):645-8.

2. Orsama AL, Mattila E, Ermes M, van Gils M, Wansink B, Korhonen I. Weight rhythms: weight increases during weekends and decreases during weekdays. Obes Facts. 2014;7(1):36-47.

3. Bhutani S, Kahn E, Tasali E, Schoeller DA. Composition of two-week change in body weight under unrestricted free-living conditions. Physiol Rep. 2017;5(13).

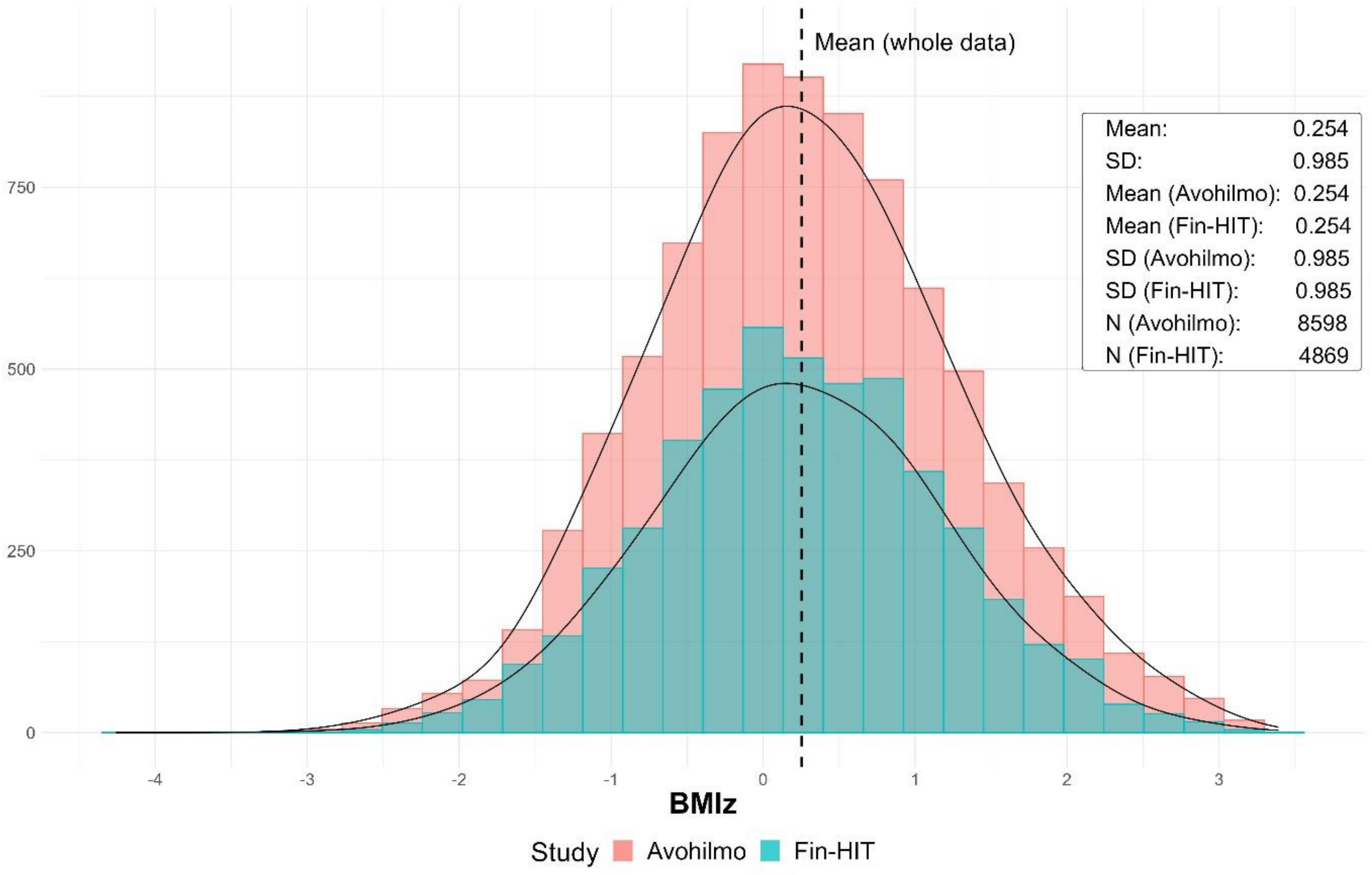

Supplementary Figure 1*. Histogram of BMIz values across data sources with the mean, standard deviation (SD), and number of observations. Data includes individuals for whom measurements in both sources are available: Avohilmo (n = 8598) and Fin-HIT baseline and follow-up (n = 4869).*

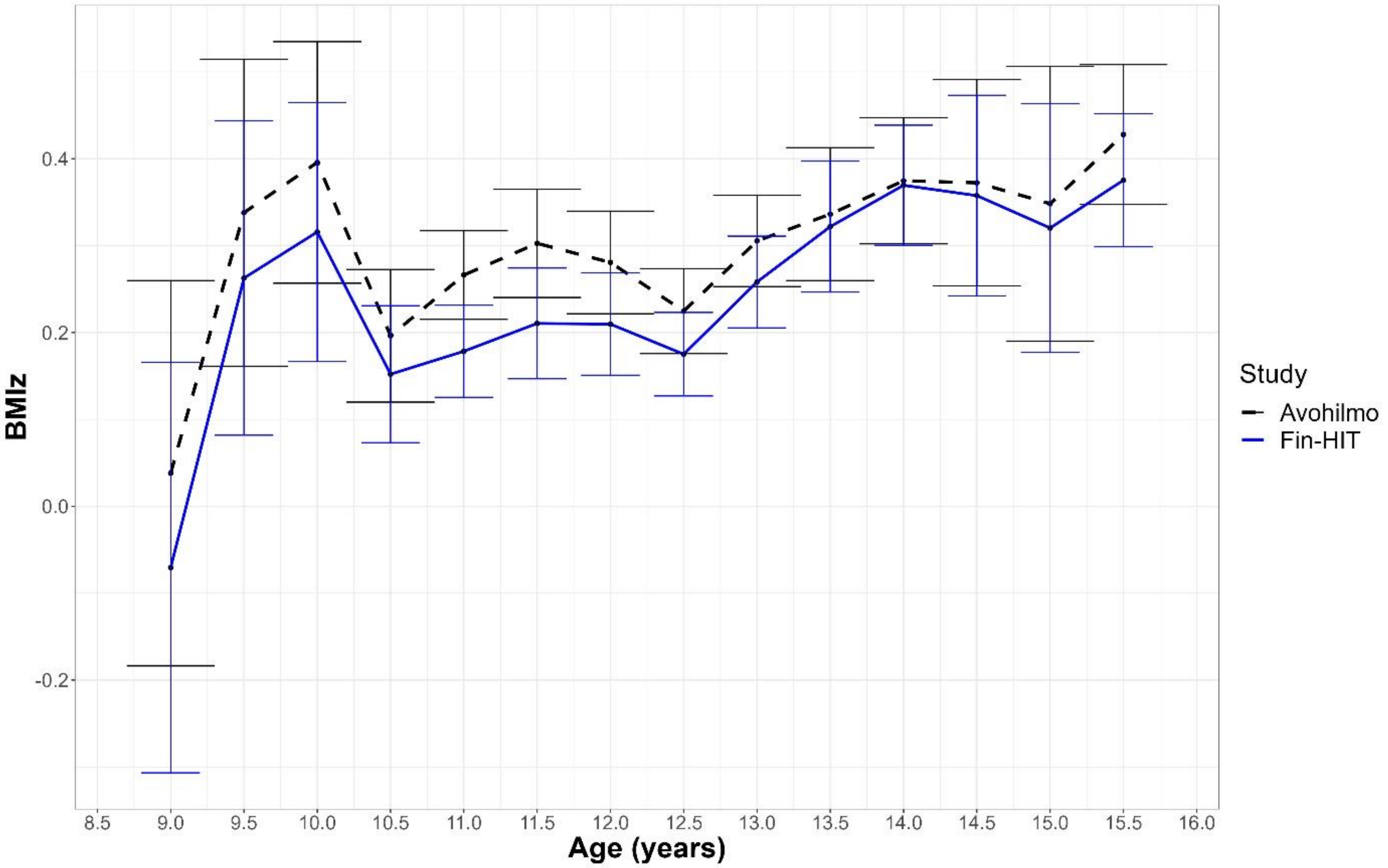

Supplementary Figure 2. *Comparison of paired data with the mean BMIz values and the standard error of the mean (SEM) between the Avohilmo and Fin-HIT datasets by age category.*

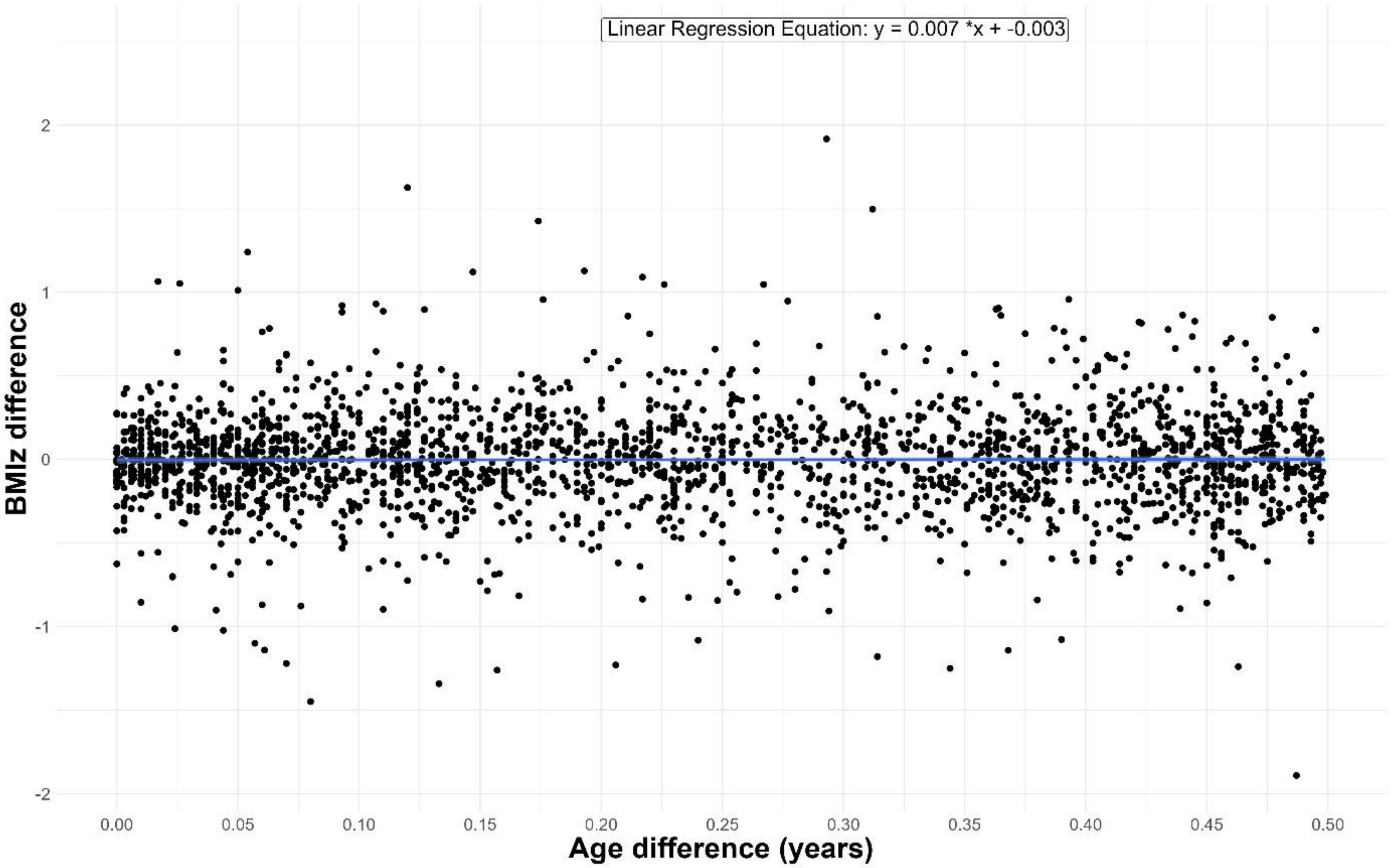

Supplementary Figure 3. *Intraindividual differences in BMIz remain constant regardless of the time difference between measurements (from 0 to 6 months).*

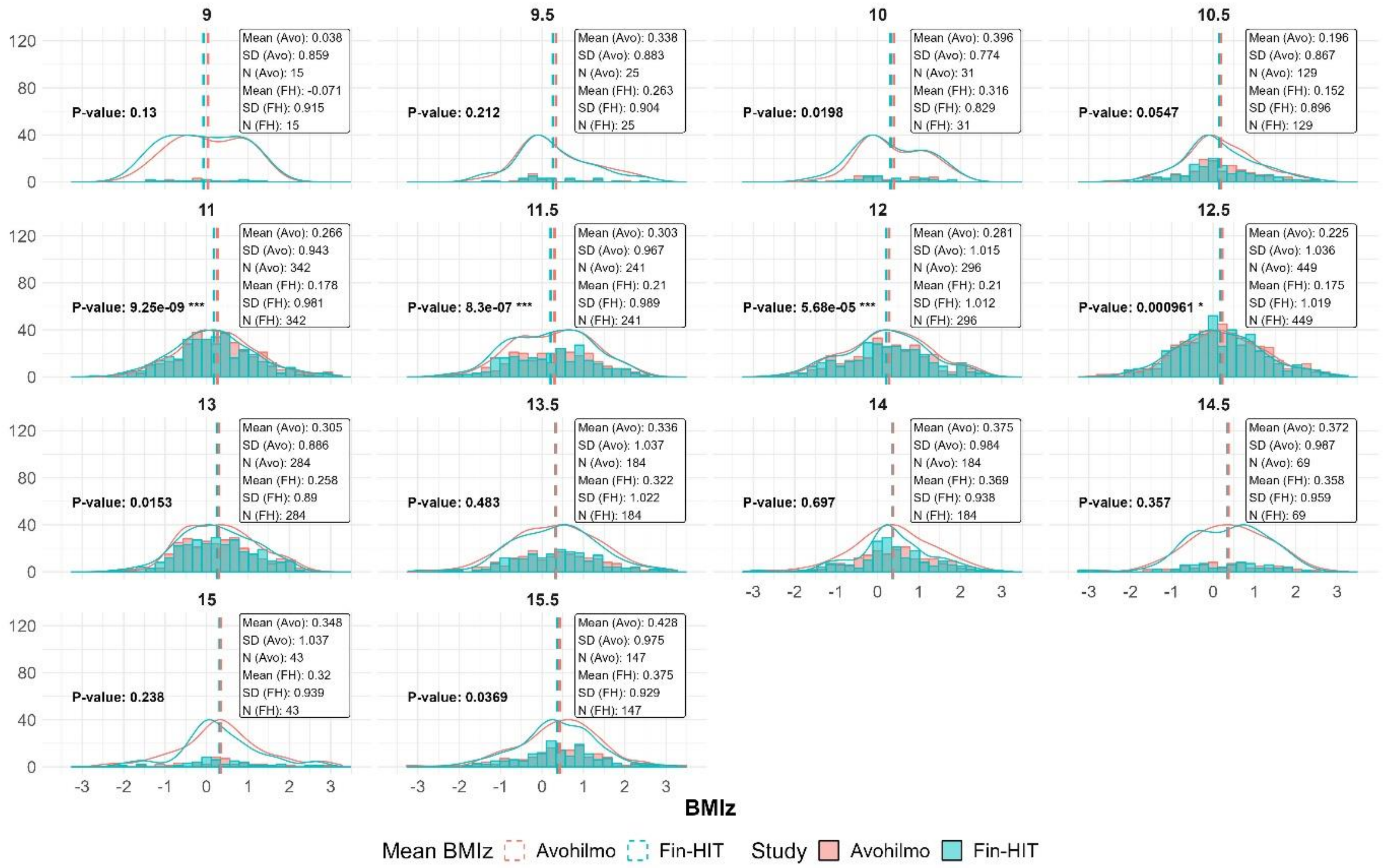

Supplementary Figure 4. *Comparison of the BMIz distributions between paired Avohilmo and Fin-HIT measurements per age category. Statistically significant differences were observed in the 11.0-, 11.5-, 12.0-, and 12.5-year age groups using paired sample t-tests. The paired sample t-test was also employed for age categories 9.0, 10.5, 13.0 and 13.5, while the Wilcoxon signed-rank test was utilised for age categories 9.5, 14.0, 14.5, and 15.5. A Bonferroni correction (0.05 / 14 = 0.00357) was applied to adjust the p value threshold for statistical significance. Inside the textboxes, Avohilmo is abbreviated as 'Avo' and Fin-HIT as 'FH'.*
